# CORE_TF: a user-friendly interface to identify evolutionary conserved transcription factor binding sites in sets of co-regulated genes

**DOI:** 10.1186/1471-2105-9-495

**Published:** 2008-11-26

**Authors:** Matthew S Hestand, Michiel van Galen, Michel P Villerius, Gert-Jan B van Ommen, Johan T den Dunnen, Peter AC 't Hoen

**Affiliations:** 1 The Center for Human and Clinical Genetics, Leiden University Medical Center, Postzone S4-0P, PO Box 9600, 2300 RC Leiden, The Netherlands

## Abstract

**Background:**

The identification of transcription factor binding sites is difficult since they are only a small number of nucleotides in size, resulting in large numbers of false positives and false negatives in current approaches. Computational methods to reduce false positives are to look for over-representation of transcription factor binding sites in a set of similarly regulated promoters or to look for conservation in orthologous promoter alignments.

**Results:**

We have developed a novel tool, "CORE_TF" (Conserved and Over-REpresented Transcription Factor binding sites) that identifies common transcription factor binding sites in promoters of co-regulated genes. To improve upon existing binding site predictions, the tool searches for position weight matrices from the TRANSFAC^*R *^database that are over-represented in an experimental set compared to a random set of promoters and identifies cross-species conservation of the predicted transcription factor binding sites. The algorithm has been evaluated with expression and chromatin-immunoprecipitation on microarray data. We also implement and demonstrate the importance of matching the random set of promoters to the experimental promoters by GC content, which is a unique feature of our tool.

**Conclusion:**

The program CORE_TF is accessible in a user friendly web interface at . It provides a table of over-represented transcription factor binding sites in the users input genes' promoters and a graphical view of evolutionary conserved transcription factor binding sites. In our test data sets it successfully predicts target transcription factors and their binding sites.

## Background

There are both experimental and computational approaches to identify transcription factors (TF) and their relevant binding sites. In the wet lab, hypothesis driven techniques, such as deletion constructs with luciferase reporter assays and chromatin-immunoprecipitation on microarrays (ChIP-on-chip), can be used to identify TF binding site (TFBS) regions. Luciferase assays can prove that a specific region has regulatory function, but is laborious and time consuming. ChIP-on-chip is more global, but requires prior knowledge of which TF to target using a specific antibody and is laborious, time consuming, and expensive. Faster and cheaper *in silico *methods have been in development which can identify potential TF and their binding sites. They also tend to target more precise the TFBS instead of just containing a TFBS region. However, finding TFBS can be extremely difficult since they may be less than 12–14 bp long and their consensus binding sites may be fairly loose [1].

One method to identify TFBS for known TF is using position weight matrices (PWM) [2]. PWM summarize experimental information on the sequence preference of TF. TRANSFAC [3,4] is the leading PWM database for TFBS with 834 matrices in total (release 11.4, December 2007), compared to 123 in JASPAR [5,6].

An additional method to look for new (*de novo*) TFBS is by searching for conservation between orthologous promoters [7]. This is based on the presumption that functional elements are evolutionary conserved since mutations to such elements could be detrimental to the organism [7,8].

However, both the sequence conservation-based and the PWM approach alone produce many false positives and false negatives. We therefore created CORE_TF, a program using both methods to reduce false predictions. We first look for TF involved in a biological process of interest, relying on the presumption that similarly expressed genes have common TF as regulators. To do this, and reduce false predictions with PWM, we search for TFBS that occur more often in a co-regulated set of promoters compared to random promoters. This algorithm, in analogy to the work of Elkon et al, 2003 [9], implements a binomial test to evaluate for this over-representation. Some PWM have a bias towards certain nucleotides, such as T's and A's for a TATA box binding TF and would therefore likely be over-represented if an experimental set had high numbers of T's and A's and the random set had equal content of all four nucleotides. We therefore also offer the option to exclude biases based on GC content by matching random promoters with approximately equal GC content to the experimental promoters. To identify individual TFBS with increased precision, and add additional support for the relevant TF, we subsequently scan individual promoters for cross-species conservation, again employing TRANSFAC matrices. All steps are flexible allowing for a multitude of input types (Ensembl [10] gene IDs, nucleotide sequences, or selected by CORE_TF).

We also compared CORE_TF to two existing programs: oPOSSUM [11] and ConTra [12].

CORE_TF is accessible as a web-page. In this paper, we present and evaluate the performance of our web-based tool for identification of TFBS.

## Implementation

### CORE_TF construction format

**Figure 1:**
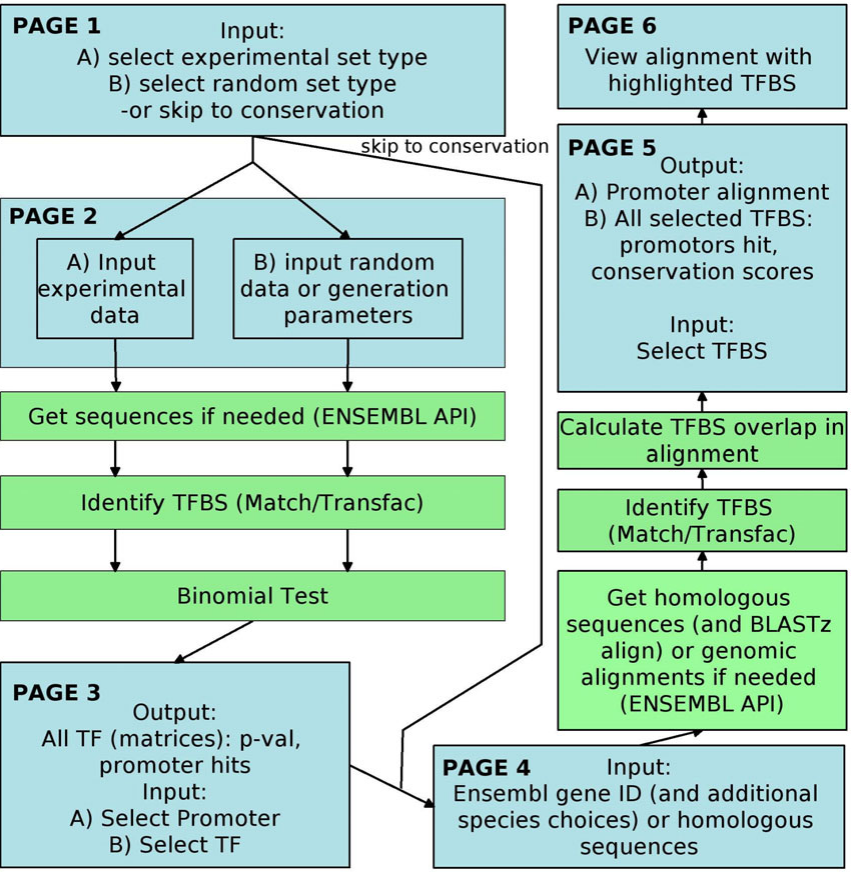
**Flowchart of CORE_TF runs**. CORE_TF runs linearly through 6 web pages. Pages 1 and 2 take as input experimental gene/promoter lists and random gene/promoter lists or requests to create random lists. Depending on format, sequences are retrieved with Ensembl API or random lists generated before identifying TFBS with Match/TRANSFAC. A binomial test is run to identify over-represented TFBS in the experimental set compared to the random set and displayed in page 3 as a table. In the table TF and a promoter can be selected which are sent to page 4. If requested homologs and sequences or genomic alignments are retrieved from Ensembl for the selected promoter. If not already a genomic alignment input sequences or retrieved sequences are aligned with BLASTz. TFBS are identified with Match/TRANSFAC, overlapping TFBS are identified and scores calculated, and the data is displayed in page 5. Conserved TFBS can be selected and displayed as highlights in the alignment in page 6.

The main script is written in Perl and presented in HTML on an Apache web-server. Input and table sorting is done using an edited Java script: sorttable.js [13]. By default, following the title page, there are 6 pages that are run in a linear fashion feeding the results of one page into the next (figure 1).

Page one allows a user to select run options and input criteria, including a p-value cut-off for highlighting data (see below), 6 different Match (the program that aligns TRANSFAC PWM to nucleotide sequences) [3,14] settings (minimize false positives, minimize false negatives, minimize the sum of both error rates, and non-redundant sets of these 3 settings), and data input type for a set of experimental promoters and a set of random promoters. The experimental promoter lists are entered as sequences in fasta format or Ensembl gene IDs. Five options are available for the random promoter list input: sequences in fasta format, an Ensembl gene ID list, randomly retrieve Ensembl promoters, pre-constructed promoter sets, and pre-retrieved sequence sets that are matched to the experimental set based on percentage of GC content. There is also an option to skip the over-representation analysis and go directly to page 4.

Depending on the selections from page 1, page 2 presents text boxes to paste in lists of fasta format sequences or Ensembl gene IDs, or radio-buttons to select a certain number of random promoters for the appropriate species, or species based check boxes for pre-constructed runs or %GC matched runs. If CORE_TF must retrieve promoters there are two options to define promoter sequences. The first option is to call a promoter as exon 1 plus a user defined number of base-pairs (bp) upstream. The second option is to define a promoter sequence as a user specified number of bp before and after the start of exon 1. The pre-constructed (approximately 3000 promoters) and pre-retrieved sets to match %GC on (approximately 10000 promoters, of which 3000 are selected) are based on 1000 bp upstream of exon 1 and exon 1 sequence.

**Figure 2:**
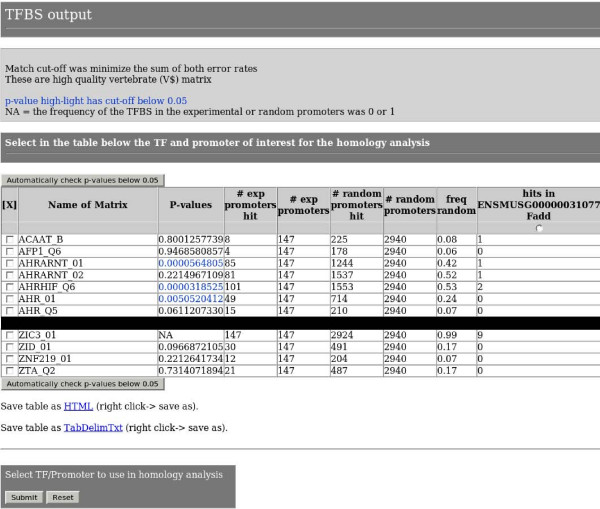
**Page 3 screen-shot**. Page 3 of CORE_TF displays the following columns: selection boxes for the next page's analysis, all TFBS matrices with hits, the p-value, the number of experimental promoters hit, the number of experimental promoters analyzed, the number of random promoters hit, the number of random promoters analyzed, frequency of hits in the random data, as well as a column for each experimental promoter analyzed indicating the number of TFBS hit in it. Our page is lengthy, so for display purposes in this figure we deleted the middle TFBS as indicated by the large black bar.

If requested, page 3 (figure 2) uses Ensembl API to retrieve promoters from a locally installed Ensembl database or from the web-based Ensembl database depending on CORE_TF installation. If the option to use %GC matched random sequences is selected CORE_TF matches pre-retrieved promoter sequences to the experimental promoter sequences so that at least 3000 similar %GC promoters are obtained. It then uses Match to scan all sequences for the presence of TRANSFAC Professional (note: web based CORE_TF is still free access to non-commercial users) vertebrate matrices passing the PWM alignment threshold provided on page 1 (pre-constructed random promoter sets also have pre-executed Match runs and initial number of hits counted). A binomial test is carried out with the Perl module Math::Cephes [15] to identify TFBS that are over-represented in the experimental set over the random set. This is displayed on the screen as a sortable table with the TFBS name, p-value (10 digits are displayed), hits and total number in the experimental and random sets, as well as the number of matrix hits in each experimental promoter. For clarity, p-values below a defined threshold from page 1 are highlighted in blue. The table can be downloaded as an HTML file or a tab-delimited text file. The user can select a number of TFBS plus a promoter of interest and continue to the next page. There is also a Java script with a button to automatically select all TFBS with a p-value below the defined threshold.

Page 4 gives the user the opportunity to use Ensembl defined orthologs or aligned genomic regions in a selection of species (currently *H. sapiens, P. troglodytes, M. musculus, R. norvegicus, B. taurus, C. familiaris*, and *G. gallus*) or enter user defined orthologous sequences in fasta format. There is also the option to define promoters as was done in page 2. If the user skipped over-representation analysis there is a list of TFBS to chose from for analysis, otherwise CORE_TF uses TFBS selection from page 3.

**Figure 3:**
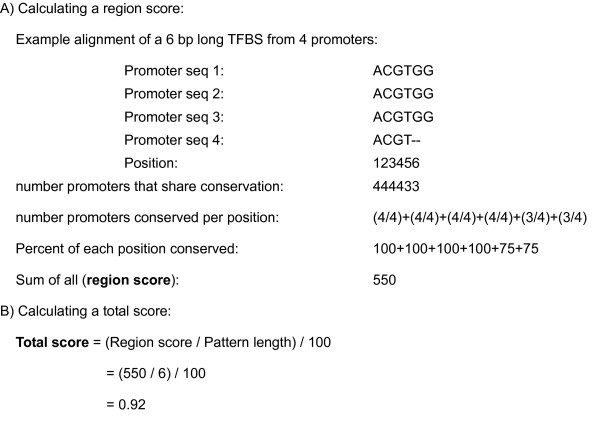
Formulas for conservation scores.

This is given to page 5 which, if necessary, retrieves either orthologous IDs and sequences or aligned genomic regions with Ensembl API. Aligned genomic regions are pairwise alignments, but CORE_TF places them into a multi-species viewed alignment. Sequences are again scanned by Match and TRANSFAC. If Ensembl genome alignments were not used, the first sequence entered or the ID used for orthologous retrieval is used as the reference to carry out a promoter sequence alignment with BLASTz [16]. Alignments are displayed on the screen. Tables are shown with each TFBS selected and the following information: total score, region score, number of promoters aligned at that point, and the length of the binding site. The region score is defined by taking the sum of 100 times the percent of each nucleotide aligned (figure 3a). The total score is defined as the region score divided by the pattern length divided by 100 (figure 3b). More specific details of these region numbers are displayed on additional tables lower in the page. The user may select a TF and submit this to the final page.

**Figure 4:**
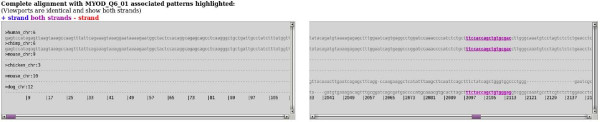
**Page 6 screen-shot of a conserved MyoD TFBS in the LAMA4 promoter**. Page 6 of CORE_TF displays two identical boxes containing aligned promoters with conserved matrices highlighted by color; blue if on the positive strand, purple if on both strands, and red if on the negative strand. If requested in the previous page to show run details (not shown in this figure), boxes with score construction for all conserved TFBS are also displayed, as well as the patterns of all selected matrices hit. Here we show an example of a MyoD TFBS (matrix MyoD_Q6_01) in the LAMA4 promoter conserved in human, chimp, and dog on both strands.

Page 6 (figure 4) allows for visualization in the alignment by displaying the alignment with selected TFBS highlighted according to the strand bound: blue (positive strand), purple (both strands), or red (negative strand). There is also evidence that some TF may preferentially bind one strand over the other [17]. It is up to the user to decide if their TF is strand specific or not.

### CORE_TF evaluation with expression and ChIP-on-chip data

To verify the performance of our algorithms we used expression and ChIP-on-chip data from Cao et al 2006 [18]. They studied the promoter binding of two major regulators of muscle differentiation (MyoD and Myog) and expression profiles in embryonic fibroblasts from MyoD/Myf5 knockout mouse transduced with a MyoD-estrogen receptor hormone binding fusion protein (termed MDER cells). These cells have been modified so that they can be studied during differentiation with or without MyoD or Myog present. Promoter binding was also studied in a common mouse myoblast cell line (C2C12).

ChIP-on-chip is a technique using a TF targeting antibody that is used to pull-down TF bound DNA fragments, which are then amplified, labeled, and hybridized to a (promoter or tiling) microarray. As a positive control set for TF binding, we took those promoters from the ChIP-on-chip data that showed enrichment for MyoD or Myog binding sites (p-value < 0.001). We re-analyzed the Affymetrix expression data by applying a RMA summarization and normalization and using the R package limma [19,20] to fit a linear model containing the following factors: MyoD expression (yes/no), Myog expression (yes/no), and time of differentiation (0, 24, 48, and 96 h). As a positive control set for MyoD or Myog-induced regulation of gene expression we took the top 200 or less genes based on the effect of MyoD or Myog expression, respectively. When needed, accession numbers were converted to Ensembl gene IDs using Idconverter [21].

For the 200 most significantly induced genes, we evaluated whether their promoters contained MyoD or Myog binding sites according to the ChIP-on-chip data. We expect that the smaller more specific lists would have a higher percent of promoters with true binding sites (significant on the ChIP-on-chip platform) and therefore likely to contain more significantly over-representation TFBS in our predictions. We found that as a general trend this is true that the smaller more specific expression lists contain a higher percent of true positives (significant ChIP-on-chip genes) (additional file 1).

### Random data size evaluation

We evaluated what would be an appropriate number of random promoters by running a set of 14 experimental promoters against several random set sizes; 500, 1000, 2000, and 4000. For this, the Match cutoff was set to minimize the sum of false positives and negatives. For this test we used a promoter size of 1000 bp before exon 1 and all of exon 1. The larger the random size used the more consistent the number of TFBS that were identified (additional file 2), but also the longer the run time. We found a random size of 2000 promoters to be the best trade off between accuracy and speed.

### Promoter size evaluation

We evaluated an appropriate promoter size for our transcription factors of interest by taking the Cao et al 2006 expression data top 50 MyoD or Myog promoters for the appropriate induction (MyoD or Myog) compared to 2000 purely random mouse Ensembl promoters. We varied the promoter size to include exon 1 plus an additional number of bp upstream; 500, 1000, 2000, and 4000. Analysis showed that with a Match setting to minimize false positives a promoter size of 2000 bp + exon 1 was best, whereas with a Match setting to minimize the sum of false positives and negatives a promoter size of 1000 bp + exon 1 was preferable (additional file 3). We continued with a Match setting to minimize the sum of false positives and negatives setting using 1000 bp upstream + exon 1 as our promoter size.

### Evaluation of GC content

To evaluate the effect of GC content we ran purely random Ensembl promoters (the FAST setting of CORE_TF) on all Cao et al ChIP data. We then compared that to runs with the option to get random promoters of approximately equal %GC content compared to the experimental set (the Similar %GC option).

### Wet-lab verification of a CORE_TF predicted conserved TFBS

To give wet-lab confirmation to the results of the CORE_TF conservation predictions we used the TransFactor kit with double stranded DNA designed on a LAMA4 (ENSG00000112769) MyoD predicted TFBS conserved between human, chimp, and dog (figure 4). This was an Ensembl genomic alignment run with a Match setting to minimize the sum of false positives and false negatives. The promoter size was defined as 3000 bp upstream of exon 1 and including exon 1. We also included a negative control of the same DNA sequence with four mutations. Recombinant MyoD protein was used to test for binding. For more details on the TransFactor run see the additional material (additional file 4).

### CORE_TF compared to an existing program: oPOSSUM

To evaluate our script with existing technology we ran the Cao et al 2006 expression data (most significant 20, 50, 100, and 200 genes) through the oPOSSUM website [11]. We chose oPOSSUM for comparison since it performs similar analysis and is freely available. We used their custom single site analysis page. Other than setting to mouse, vertebrate JASPAR matrices, retrieving 1000 bp up and 433 bp downstream (using Ensembl API we calculated this as the average size of exon 1) of the transcription start site, and showing all results, all settings used their defaults. It must be noted that JASPAR only has a matrix for Myf, which represents a TF family including MyoD and Myog. We also used their number of hits in their background and target genes to run a binomial test in the statistical package R to match our data.

### CORE_TF compared to an existing program: ConTra

We also chose to evaluate CORE_TF versus an additional easily viewable cross-species conservation program, ConTra [12]. As a test promoter for comparison we used the LAMA4 (ENSG00000112769) promoter, for which we had a lab verified MyoD TFBS. The ConTra website was run on all default parameters (selecting transcript ENST00000230538), except for looking at 3000 bp upstream instead of 2000 bp upstream (giving a promoter the same size as the CORE_TF run). We looked at the PWM MyoD_Q6_01. This was the only PWM for MyoD available at the ConTra website and the best performing for CORE_TF with this promoter.

## Results and discussion

### CORE_TF work flow and function

We have developed a series of web pages to identify TFBS in two sequential processes. First, pages 1 to 3 allow a user to predict TF that regulate a set of co-regulated genes. This is done by identifying TFBS that are over-represented in the promoters of an experimental (e.g. similar expressed genes from microarray data) compared to a random data set, taking GC content into account if requested. These results are displayed in a sortable table in page 3 (figure 2). Secondly, pages 4 to 6 allow a user to identify specific TFBS by looking for across species conservation of TFBS selected from the TFBS in page 3 and the promoters of page 3. This is done on Ensembl genomic alignments or BLASTz alignments of orthologous promoters provided by Ensembl or the user. Across species conserved TFBS are displayed in tables (calculations as in figure 3) in page 5 and as aligned promoters in a graphical format (figure 4) in page 6.

Alternatively, if a user did not wish to look at a list of promoters, but just a single promoter they could look purely for cross-species conserved TFBS by skipping straight to page 4 from page 1. They must then provide which promoter they want to search and a set of TFBS from a web displayed list. In theory they could paste the sequences conserved in the alignments back into the over-representation pages to find TFBS over-represented in conserved regions (as opposed to the normal order of looking for conservation with over-represented TFBS).

### Prediction of over-represented TFBS

To evaluate the performance of our tool we first used the Cao et al 2006 ChIP-on-chip data as a positive control. We tested whether the promoters in the ChIP pull-down were enriched for the binding sites for the transcription factors targeted in the ChIP experiments compared to a random set of promoters. To evaluate the effect of matching promoters for %GC content, CORE_TF was run with a purely random selected set of promoters (FAST option) and a random set of promoters with matched %GC content as controls (similar %GC option). Using both sets of random promoters, CORE_TF found a significant over-representation (p-value < 0.05, after applying multiple test correction with Benjamini & Hochberg in R [22]) for the MyoD matrix MYOD_Q6 in the MyoD bound promoters and the Myog matrix MYOGENIN_Q6 in the Myog bound promoters, in both C2C12 and MDER cells (additional file 5). The MyoD matrix MYOD_Q6_01 was also significant in all MyoD targeted runs except the MDER MyoD with random promoters matched on %GC content.

Strikingly, by ranking TFBS on p-value, we demonstrate that the target transcription factors were higher ranked with the %GC matched promoters as control rather than with the purely random set of control promoters (table 1), indicates that improper matching of GC content leads to false positive identification of TFBS. By evaluating the distribution of p-values for all TF using both random sets, we observed purely random promoters yield more high and low p-values than a random set of promoters matched on %GC content (additional file 6). Since our target ChIP TF remained significant when using %GC matched promoters, resulting in a smaller list of significant TFBS, we believe this method to yield less false positives.

**Table 1:** Cao et al 2006 top ChIP-on-chip predictions with CORE_TF

A. MyoD ChIP-on-chip							
C2C12 MyoD FAST	p-val*	C2C12 MyoD %GC	p-val*	MDER MyoD FAST	p-val*	MDER MyoD %GC	p-val*
AP1_Q6_01	0	MYOGENIN_Q6	1.5E-06	AP1_Q6_01	0	AP4_Q5	0
E2F1DP1_01	0	AP4_Q5	2.3E-06	AP4_Q5	0	AP4_Q6	3.1E-06
E2F4DP2_01	0	E2A_Q2	2.7E-06	COUP_DR1_Q6	0	MYOGENIN_Q6	2.7E-05
E2F_Q4	0	AP4_Q6	8.8E-06	E2F1DP1_01	0	AP4_Q6_01	2.7E-04
E2F_Q6_01	0	**MYOD_Q6**	8.8E-06	E2F4DP2_01	0	AP4_01	1.2E-03
GATA3_01	0	AP4_Q6_01	5.1E-05	E2F_Q4	0	**MYOD_Q6**	4.5E-03
MAF_Q6_01	0	E47_01	1.1E-03	E2F_Q6_01	0	E2A_Q2	5.2E-03
NF1_Q6_01	0	E12_Q6	1.4E-03	MAF_Q6_01	0	LBP1_Q6	2.3E-02
NFE2_01	0	LBP1_Q6	4.0E-03	NF1_Q6_01	0	HEN1_02	7.6E-02
OSF2_Q6	0	E2A_Q6	4.6E-03	NFE2_01	0	TAL1BETAE47_01	7.6E-02
AP4_Q5	7.9E-09	SMAD_Q6_01	2.7E-02	NFKB_Q6	0	**MYOD_Q6_01**	1.7E-01
MYOGENIN_Q6	7.9E-09	**MYOD_Q6_01**	4.4E-02	OSF2_Q6	0	HEB_Q6	2.9E-01
LBP1_Q6	2.2E-08	AP1FJ_Q2	4.5E-02	AP4_Q6	3.5E-09	HELIOSA_01	2.9E-01
AP4_Q6	5.8E-08	AP1_Q4	5.8E-02	GATA3_01	3.5E-09	AP1_Q4	4.0E-01
AP4_Q6_01	6.2E-08	E47_02	9.4E-02	LBP1_Q6	6.5E-08	HNF4_01	4.0E-01

B. Myog ChIP-on-chip							

C2C12 Myog FAST	p-val*	C2C12 Myog %GC	p-val*	MDER Myog FAST	p-val*	MDER Myog %GC	p-val*
AP1_Q6_01	0	AP4_Q5	0	AP1_Q6_01	0	AP4_Q5	0
AP4_Q5	0	AP4_Q6	0	AP4_Q5	0	AP4_Q6	0
AP4_Q6	0	**MYOGENIN_Q6**	0	AP4_Q6	0	**MYOGENIN_Q6**	0
E2F1DP1_01	0	MYOD_Q6	5.0E-06	COUP_DR1_Q6	0	AP4_Q6_01	2.5E-06
MAF_Q6_01	0	AP4_Q6_01	1.1E-05	E2F1DP1_01	0	LBP1_Q6	8.0E-06
**MYOGENIN_Q6**	0	E2A_Q2	9.0E-04	E2F4DP2_01	0	MYOD_Q6	8.1E-06
NF1_Q6_01	0	AREB6_01	6.9E-03	E2F_Q4	0	E2A_Q2	7.1E-03
NFE2_01	0	MYOD_Q6_01	1.4E-02	E2F_Q6_01	0	MYOD_Q6_01	8.6E-03
OSF2_Q6	0	LBP1_Q6	1.4E-02	LBP1_Q6	0	CLOCKBMAL_Q6	4.5E-02
E2F4DP2_01	4.7E-09	AP4_01	1.9E-02	MAF_Q6_01	0	AP2ALPHA_01	5.7E-02
COUP_DR1_Q6	1.6E-07	AP1_Q4	2.2E-02	**MYOGENIN_Q6**	0	ZEC_01	5.8E-02
AP4_Q6_01	2.3E-07	ZEC_01	2.2E-02	NF1_Q6_01	0	AP2_Q6	7.7E-02
E2F_Q4	1.3E-06	E2A_Q6	7.1E-02	NFE2_01	0	AP4_01	1.1E-01
GATA3_01	6.5E-06	ATF6_01	8.0E-02	OSF2_Q6	0	PPARG_01	1.2E-01
MYOD_Q6	7.5E-06	E47_01	8.2E-02	AP4_Q6_01	6.4E-09	CMYC_02	1.2E-01

CORE_TF predictions on Cao et al 2006 ChIP-on-chip data. Target TFBS are presented in bold. * = p-values are Benjamini & Hochberg corrected. Note: in the MyoD FAST runs MYOD_Q6 and MYOD_Q6_01 had p-values < 0.05 but were not in the top 15 significant TFBS.

**Figure 5:**
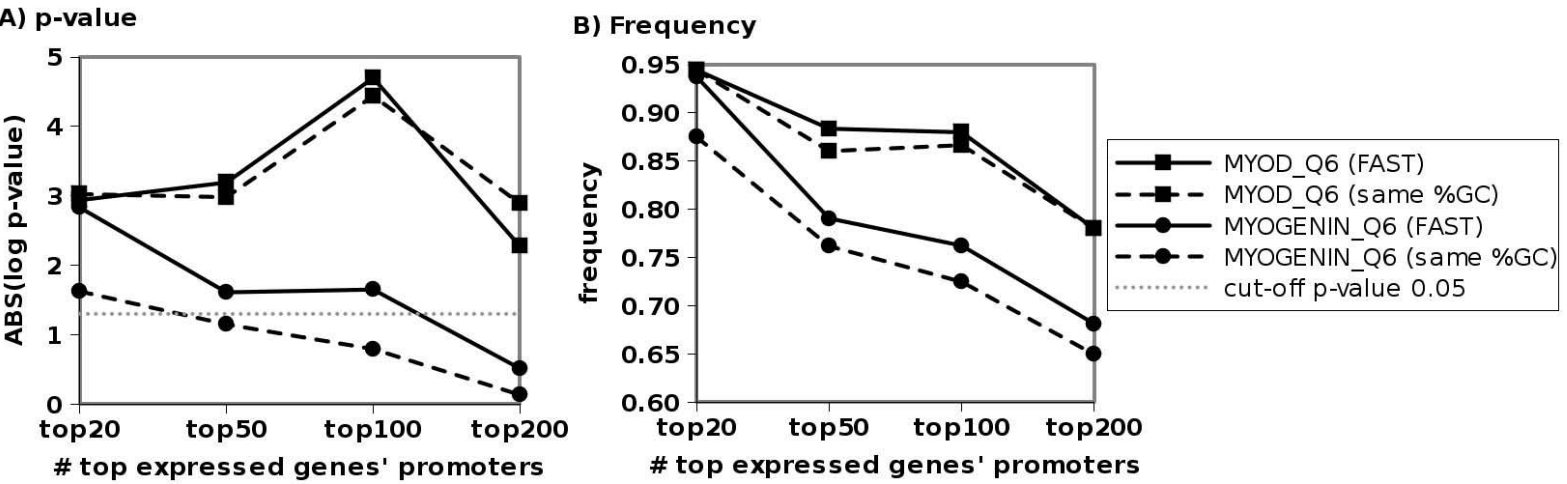
**Significance of myogenic TFBS in expression data**. The (A) significance (as the absolute value of the log_10 _p-value) and (B) frequency of MyoD (matrix MyoD_Q6) or Myog (matrix MYOGENIN_Q6) TFBS in varying number of promoters from genes with increasingly less significant differences in expression upon MyoD or Myog activation are shown. As would be expected, the smaller more significant lists generally have higher frequency and more significant p-values than larger less specific lists.

To demonstrate that our algorithm is able to find shared regulatory sites in co-regulated genes identified in expression microarray data we evaluated whether genes for which the expression level increased upon MyoD or Myog activation were enriched for MyoD or Myog binding sites. We ran sets consisting of the 20, 50, 100, and 200 genes most significantly affected by MyoD or Myog activation versus a random set of approximately equal %GC content (additional file 7). We found significant enrichment of the MyoD_Q6 matrix in all MyoD enriched sets. We also found MYOD_Q6_01 enriched in the top 50 and top 100 MyoD enriched sets. MYOGENIN_Q6 was found enriched in the top 20 Myog enriched set only. Other PWMs for MyoD or Myog and other sets of promoters were not significant or considered "NA" due to 100% of promoters hit in the experimental data. The same data was also run through with the CORE_TF FAST setting. We found that the two settings perform similar, with slightly higher frequencies but slightly less significant p-values when matching on %GC (Figure 5). Additionally, as expected the smaller more specific lists generally have higher frequencies and lower p-values than larger, less specific lists (Figure 5).

### Orthologous alignments versus genomic alignments

In many CORE_TF runs we assessed the conserved TFBS using alignments based on homologous Ensembl promoters as well as Ensembl genomic alignments. Ensembl pairwise alignments can be considered syntenic (they are grouped to make the actual Ensembl synteny blocks) [23]. Ensembl orthologs are identified using protein tree calculations [10]. The number of promoters aligning and the quality of the alignment to the reference promoter varies tremendously amongst different promoters for both methods (data not shown), but we did not find one method outperforming the other. Synteny does not imply the start of one gene corresponds to the start of a gene in another species. Therefore, this could give poor predictions for TF that bind and function close to the transcription start site. However, due to many incorrect exon 1 annotations it is also possible that using orthologous promoter alignments may align regions that are not corresponding regions (if an annotation missed exon 1, exon 2 would be annotated as exon 1 and we would instead align to it). Therefore there is not one alignment method that outperforms another to predict conserved TFBS.

### TFBS conserved in orthologous alignments

The top 10 ranked genes of the Myog-induced genes were inspected for the presence of MYOGENIN_Q6 motifs. To this end, all available orthologs for the mouse genes were retrieved. All conserved TFBS and their conservation scores are reported in Table 2. There are seven promoters which appear to have conserved binding sites. Four of these promoters (Chrng, Myog, Acta1, and Tnnc1) had hits in three or more orthologs. We also inspected the MyoD induced genes presence of MyoD_01 motifs using the same approach and identified two promoters with conserved TFBS (Table 2). Only one promoter was found conserved over three or more orthologs (Rgs16). In addition, of the nine across species conserved TFBS all except Tnnc1 (not on the array), Tnnc2, Rgs16, and Nptx1 were found significant in the ChIP-on-chip data. Literature was examined to see if predictions were correct. We found evidence for binding of Myog to Myog [24], Tnni1 [25], and Chrng [26]. We also found evidence for MyoD binding Nptx1, also called NP1 [27].

**Table 2:** Orthologous conservation of target TFBS in target genes

A						
Gene Name	GeneID	TF Name	Tot. Score	Score	#Promos	Length
Chrng	ENSMUSG00000026253	MYOGENIN_Q6	1	1000	5	10
Chrng	ENSMUSG00000026253	MYOGENIN_Q6	1	1000	5	10
Tnnt3	ENSMUSG00000061723	MYOGENIN_Q6	1	1000	2	10
Tnnc2	ENSMUSG00000017300	MYOGENIN_Q6	1	800	2	8
Tnni1	ENSMUSG00000026418	MYOGENIN_Q6	1	800	2	8
Myog	ENSMUSG00000026459	MYOGENIN_Q6	0.83	666.7	5	8
Acta1	ENSMUSG00000031972	MYOGENIN_Q6	0.8	640	4	8
Tnnc1	ENSMUSG00000021909	MYOGENIN_Q6	0.72	720	4	10
Acta1	ENSMUSG00000031972	MYOGENIN_Q6	0.6	480	3	8
B						
Gene Name	GeneID	TF Name	Tot. Score	Score	#Promos	Length
Rgs16	ENSMUSG00000026475	MYOD_01	1	1200	4	12
Rgs16	ENSMUSG00000026475	MYOD_01	0.5	600	2	12
Nptx1	ENSMUSG00000025582	MYOD_01	0.4	840	2	21
Nptx1	ENSMUSG00000025582	MYOD_01	0.4	480	2	12

Conserved TFBS for (A) Myog (matrix MYOGENIN_Q6) and (B) MyoD (matrix MYOD_01) from target genes' promoters in expression data. Total score represents a score of conservation from 0 to 1 over the conserved TFBS length. Score represents an additive score over the binding site. # Promos is the number of promoters with the conserved TFBS. Length is the length of the TFBS.

### Wet-lab conformation of a CORE_TF predicted conserved TFBS

To confirm a CORE_TF conserved TFBS in the lab we looked at a MyoD predicted TFBS in the LAMA4 promoter. Using Ensembl defined genomic alignments we found the matrix MyoD_Q6_01 conserved in human, chimp, and dog (figure 4). Using a recombinant MyoD protein and the TransFactor kit we found significant (p-value 1.5E-35) binding to our target TFBS compared to a mutated one (additional file 4).

### CORE_TF compared to existing programs: oPOSSUM

We compared the performance of CORE_TF (using a random set with similar %GC) to oPOSSUM, a webtool with similar objectives as ours. oPOSSUM looks for over-represented JASPAR PWM in pre-defined species alignments, but is limited to specific species alignments (e.g. human-mouse) and use of the smaller JASPAR PWM database. We used the previously mentioned expression microarray datasets for the evaluation of both programs performances. Our runs on the oPOSSUM website showed that our binomial test performs similar to their Fisher test (additional file 8). Unlike our frequency observations, the frequency identified by oPOSSUM of TFBS hits in the MyoD induced set did not show the expected high to low pattern (additional file 9). When comparing p-values from the binomial tests for the predictions by the two programs, we see similar patterns between the two programs across the top 20, 50, 100, and 200 genes, but CORE_TF has more significant MyoD predictions and oPOSSUM has more significant Myog predictions (additional file 9). It must be noted that we are only comparing over-represented TFBS whereas oPOSSUM has already taken conservation into their program at this point which may explain higher sensitivity for Myog promoters. We instead do this on individual promoters and display it graphically in the next step. We believe this graphical representation to be more interpretable.

Since we can do better in one out of two tested TF without our orthologous promoter conservation we believe CORE_TF to be a superior tool. The two programs differ on several other levels. oPOSSUM only takes Ensembl IDs as input, whereas we also accept nucleotide sequences. We also offer a larger choice of random data sets and conservation methods, as well as the choice to account for GC content. In addition, our number of vertebrate species available is six, all of which can be compared together. oPOSSUM only accepts two species comparisons at a time. For vertebrates oPOSSUM is limited to only human and mouse, both of which are in CORE_TF. In addition, we display our across-species TFBS in a graphical format, whereas oPOSSUM presents their data in a less intuitive tabular format.

### CORE_TF compared to an existing program: ConTra

We also evaluated CORE_TF versus ConTra using the LAMA4 promoter, for which we had experimental data available, as an example. ConTra is a website to identify and easily view conserved TFBS in a single cross-species promoter alignment, but cannot look for over-representation in a large promoter set. We found that in CORE_TF genomic alignment predictions there were three MyoD sites conserved between human and chimp and one TFBS conserved between human, chimp, and dog (figure 4). ConTra found the same sites, but also three additional (additional file 10 and data not shown). Two of the three human/chimp CORE_TF conserved TFBS and the human/chimp/dog CORE_TF conserved TFBS were also found conserved in the macaque in ConTra. CORE_TF did not search for macaque, but it is extremely similar to human and chimp so we believe it would not add much information. However, if a user wanted any Ensembl species added to CORE_TF adding an additional species to the scripts is very simple. It is not surprising the same sites were identified since both programs use Ensembl alignments and TRANSFAC matrices. ConTra does have the disadvantage of only using human as a reference genome for automated alignment retrievals, whereas CORE_TF can do this for all six species currently installed. Additionally, CORE_TF does not use an Ensembl multi-species defined alignment, but combines many Ensembl pair-wise alignments into one, allowing any number of Ensembl species to be included in one alignment. ConTra does not display strand specific binding which CORE_TF does by color coding. Additionally, ConTra does not search for over-represented TFBS in a group of promoters.

### Future efforts

An item that can be improved in the future is our evolutionary scoring algorithm, e.g. by taking into account the confidence of each nucleotide in the PWM. An additional improvement will be to analyze combinations of TFBS.

## Conclusion

We have developed a tool for identifying over-represented TFBS in promoters from co-expressed genes aided by the evaluation of cross-species conservation. CORE_TF is easy to use and displays results in tables or graphically allowing for easy interpretation of the results. Our method seems to correctly predict the presence of experimentally verified TFBS, as shown by our extensive analysis on Cao et al 2006 expression and ChIP-on-chip data and wet-lab conformation of a MyoD predicted TFBS in the LAMA4 promoter. We also show improvements over two existing programs (oPOSSUM and ConTra) with greater flexibility in input data, coverage of a larger number of species, more intuitive output, and the option to account for GC content.

Our tool is provided as a web service free to all non-commercial users.

## Availability and requirements

Project name: CORE_TF

Project home page: 

Operating system(s): Linux

Programming language: Perl (we used 5.8.4)

Other requirements: TransFac Professional (we used 11.2), BLASTz, sorttable.js, Math::Cephes (Perl module), Apache (we used 1.3.33)

License: GNU General Public License, v3 

Any restrictions to use by non-academics: none for website use, TransFac Professional license for a local install

## Authors' contributions

MH, JD, GO, and PH conceived of the primary concepts of the software. MH and MG did the primary programming and debugging. MV performed all primary installations on the web-server and helped in debugging code. MH, MG, and PH performed the software evaluation on expression and ChIP-on-chip data. Wet-lab work was done by MH. Manuscript drafting was done by MH, MG, JD, GO, and PH. All authors read and approved the final manuscript.

## Supplementary Material

Additional file 1 **Overlap of most significant expression genes in ChIP-on-chip data**. Indicated are the size of the lists for the top expressed genes and the percent of those contained in the significant ChIP-on-chip genes (true-positives). There is a trend that the smaller more selective expression gene lists contain a higher percent of true positives. Click here for file

Additional file 2 **Consistency of TF identification in different random set sizes**. Indicated are the number of TF that occur in 1, 2, or 3 out of 3 total runs. As expected, the larger the random set size (500, 1000, 2000, or 4000 promoters) the larger the consistency over runs. However, as indicated by the y-axis scale, this is not a very large effect. Click here for file

Additional file 3 **Optimal promoter size**. The p-value and frequency of promoters with size 500, 1000, 2000, and 4000 bp and exon 1 with Match settings to minimize false positives (Min_pos) or minimize the sum of false positives and negatives (Min_sum). Overall, we see a promoter of (A) 1000 bp + exon 1 works best for Min_sum runs and (B) 2000 bp + exon 1 works best for Min_pos runs. As expected, (C and D) frequency of TFBS hit increases as the promoters become larger. Click here for file

Additional file 4 **TransFactor_LAMA4_MyoD**. Set-up and data analysis of MyoD binding a LAMA4 promoter derived sequence with the TransFactor kit. Click here for file

Additional file 5 **Cao_et_al_2006_ChIP_CORE_TF**. CORE_TF run results to identify over-represented TFBS in MyoD/Myog ChIP-on-chip data. Click here for file

Additional file 6 **CORE_TF using random FAST runs vs runs with similar %GC**. It is visible that in all ChIP-on-chip data tested the runs on purely random Ensembl promoters (FAST runs) has a bias towards high and low p-values while the random set with a similar %GC follows a more normal distribution. This could account for false positives in the FAST runs. Click here for file

Additional file 7 **Cao_et_al_2006_expression_CORE_TF**. CORE_TF run results to identify over-represented TFBS in expression array data. Click here for file

Additional file 8 **oPOSSUM runs on expression data**. Custom oPOSSUM runs using the top 10, 20, 50, 100, and 200 genes from Cao et al 2006 expression data. oPOSSUM supplies (A) Fisher and (B) z-scores. (C) We also used their hits in the experimental and background data to generate a binomial test p-value similar to our program. (D) Frequency of TFBS hits overall declines as we stray from the top hits, as expected, but this is not an entirely smooth curve. Click here for file

Additional file 9 **CORE_TF vs oPOSSUM.** CORE_TF and oPOSSUM binomial test p-values for the top 20, 50, 100, and 200 genes from Cao et al 2006 expression data for over-expression (A) of MyoD or Myog in the appropriately induced cell line. We see comparable results in the top 20, 50, 100, and 200 sets, but better overall performance in oPOSSUM for Myog and in CORE_TF for MyoD. Frequency (B) of MyoD or Myog hits was also plotted. As expected, the smaller more significant lists generally have higher frequency and more significant p-values than larger less specific lists. Frequency of TFBS in the promoters was also overall higher in experimental data than random promoters as expected. The oPOSSUM MyoD frequency was the only plot that did not seem concordant. Click here for file

Additional file 10 **Identifying MyoD TFBS conserved in the LAMA4 promoter with ConTra and CORE_TF**. Many conserved sites were found identically between the two programs. Shown here is the most conserved TFBS found, a MyoD TFBS conserved between human, chimp, and dog in (B) CORE_TF and also macaque in (A) ConTra. Though found by both programs, CORE_TF also identifies the site is on both strands of the DNA. Click here for file
