# Supplementary figures and images for: CORE_TF: a user-friendly interface to identify evolutionary conserved transcription factor binding sites in sets of co-regulated genes
Source: BMC Bioinformatics. 2008 Nov 26;9:495. doi: 10.1186/1471-2105-9-495 (PMC2613159; doi:10.1186/1471-2105-9-495)

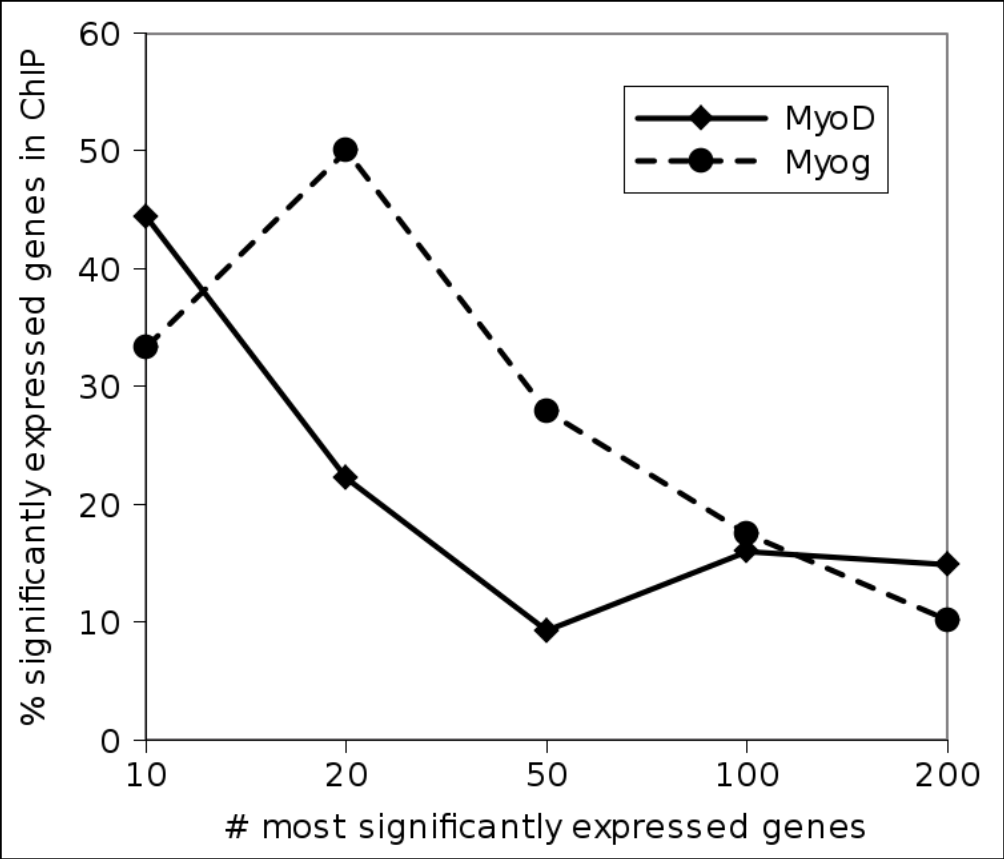

Supplement: Additional file 1 — Overlap of most significant expression genes in ChIP-on-chip data. Indicated are the size of the lists for the top expressed genes and the percent of those contained in the significant ChIP-on-chip genes (true-positives). There is a trend that the smaller more selective expression gene lists contain a higher percent of true positives. [file 1471-2105-9-495-S1.tiff]

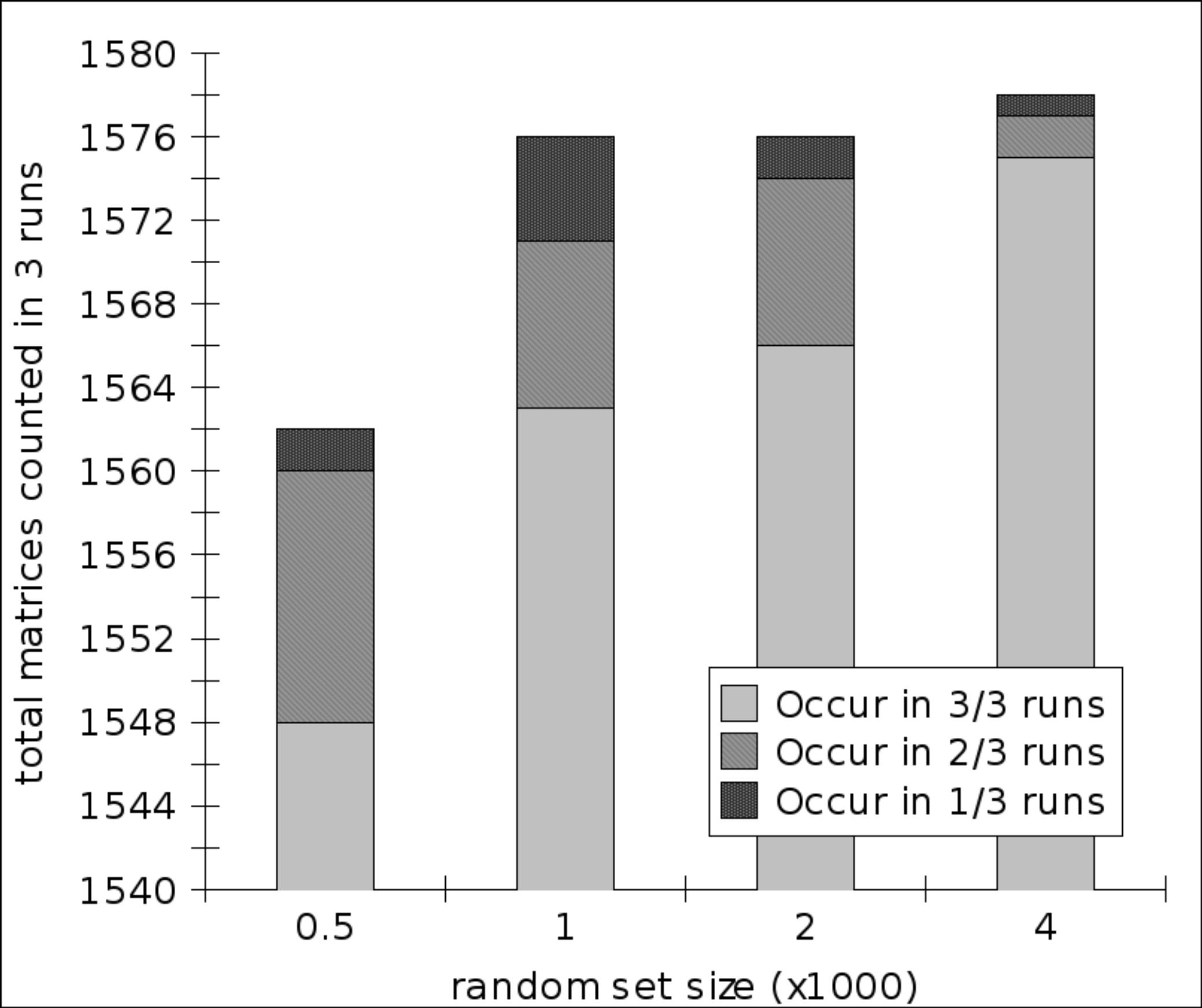

Supplement: Additional file 2 — Consistency of TF identification in different random set sizes. Indicated are the number of TF that occur in 1, 2, or 3 out of 3 total runs. As expected, the larger the random set size (500, 1000, 2000, or 4000 promoters) the larger the consistency over runs. However, as indicated by the y-axis scale, this is not a very large effect. [file 1471-2105-9-495-S2.png]

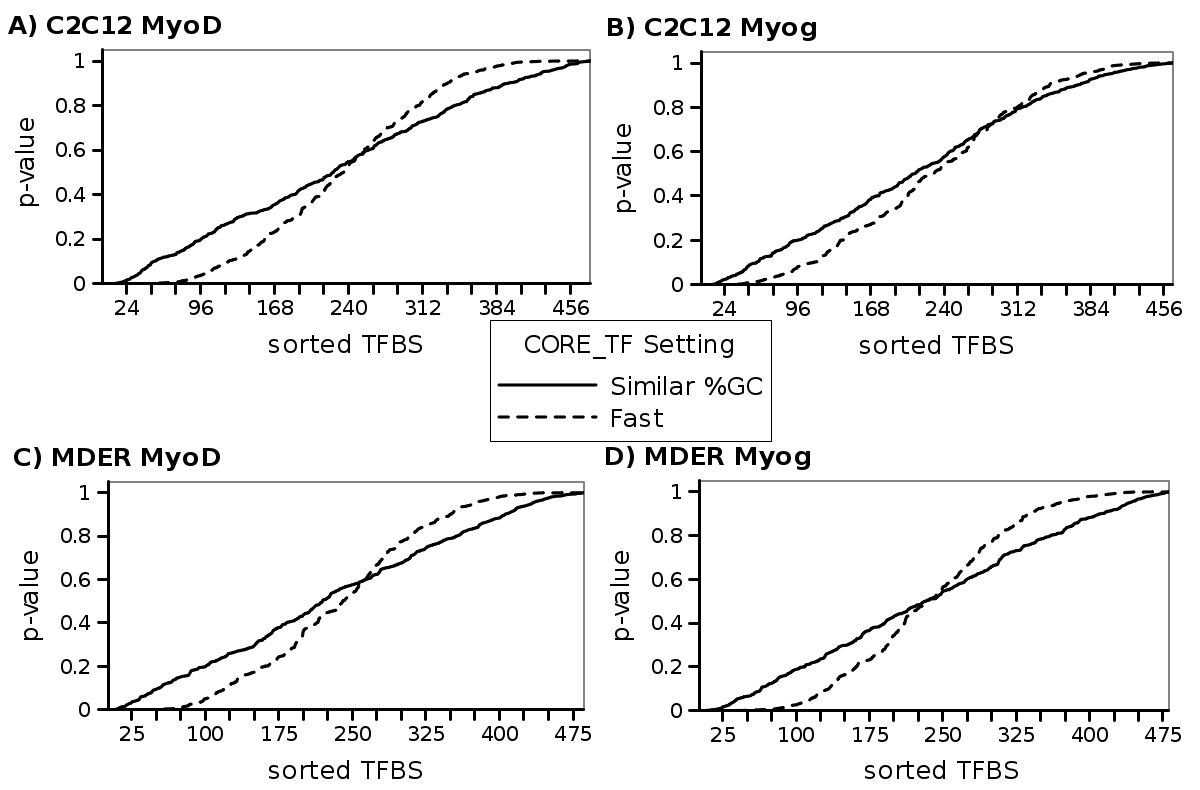

Supplement: Additional file 6 — CORE_TF using random FAST runs vs runs with similar %GC. It is visible that in all ChIP-on-chip data tested the runs on purely random Ensembl promoters (FAST runs) has a bias towards high and low p-values while the random set with a similar %GC follows a more normal distribution. This could account for false positives in the FAST runs. [file 1471-2105-9-495-S6.png]

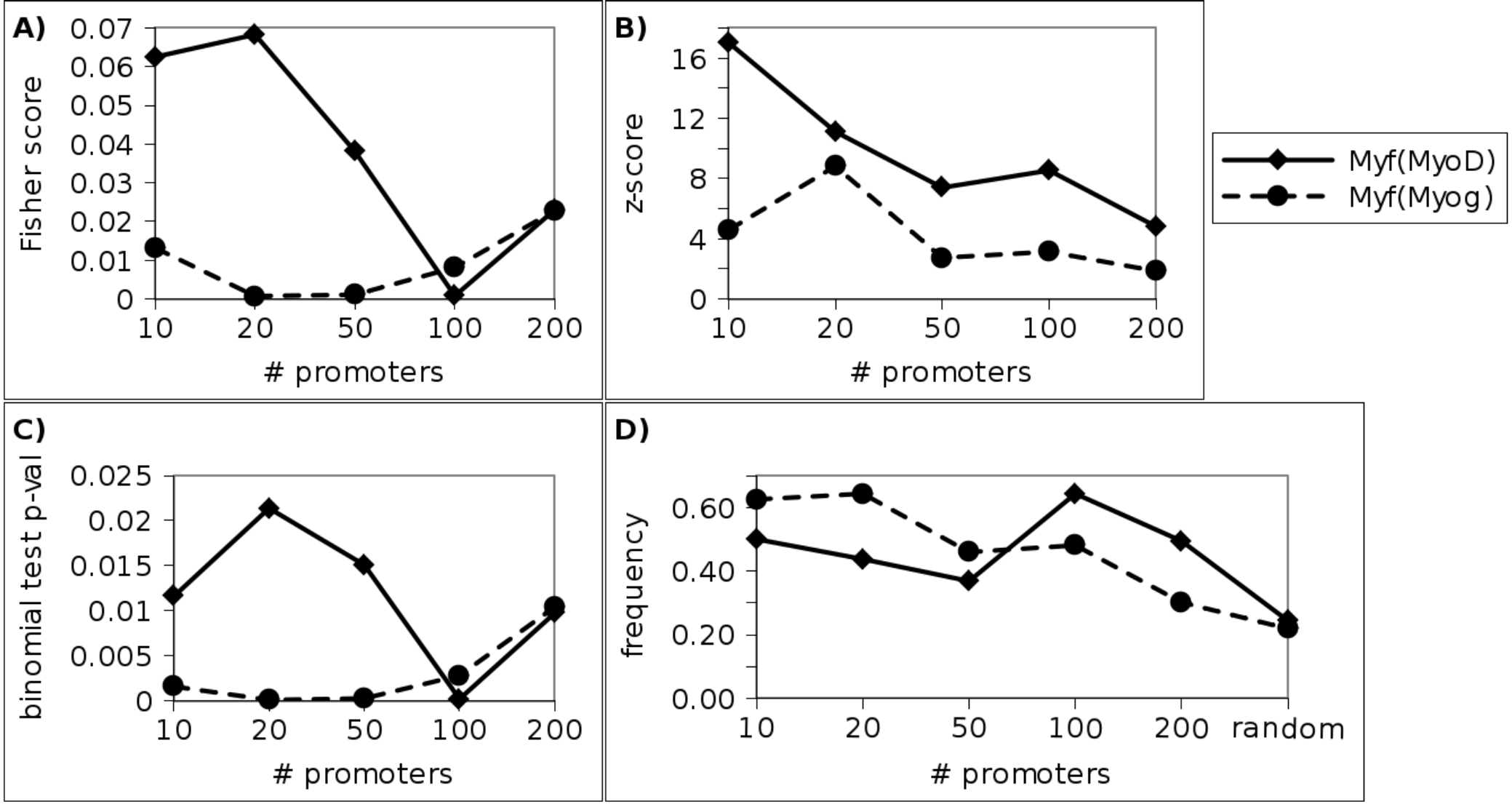

Supplement: Additional file 8 — oPOSSUM runs on expression data. Custom oPOSSUM runs using the top 10, 20, 50, 100, and 200 genes from Cao et al 2006 expression data. oPOSSUM supplies (A) Fisher and (B) z-scores. (C) We also used their hits in the experimental and background data to generate a binomial test p-value similar to our program. (D) Frequency of TFBS hits overall declines as we stray from the top hits, as expected, but this is not an entirely smooth curve. [file 1471-2105-9-495-S8.tiff]

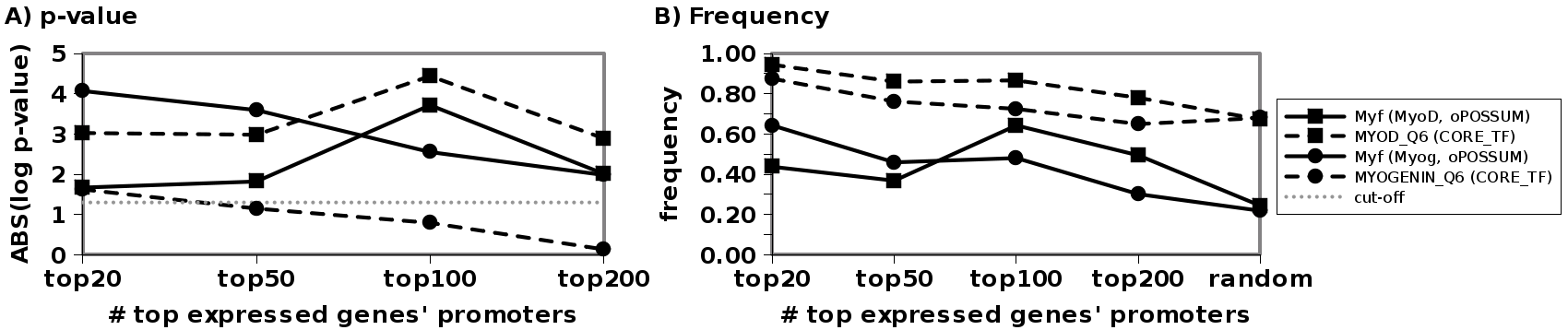

Supplement: Additional file 9 — CORE_TF vs oPOSSUM. CORE_TF and oPOSSUM binomial test p-values for the top 20, 50, 100, and 200 genes from Cao et al 2006 expression data for over-expression (A) of MyoD or Myog in the appropriately induced cell line. We see comparable results in the top 20, 50, 100, and 200 sets, but better overall performance in oPOSSUM for Myog and in CORE_TF for MyoD. Frequency (B) of MyoD or Myog hits was also plotted. As expected, the smaller more significant lists generally have higher frequency and more significant p-values than larger less specific lists. Frequency of TFBS in the promoters was also overall higher in experimental data than random promoters as expected. The oPOSSUM MyoD frequency was the only plot that did not seem concordant. [file 1471-2105-9-495-S9.png]

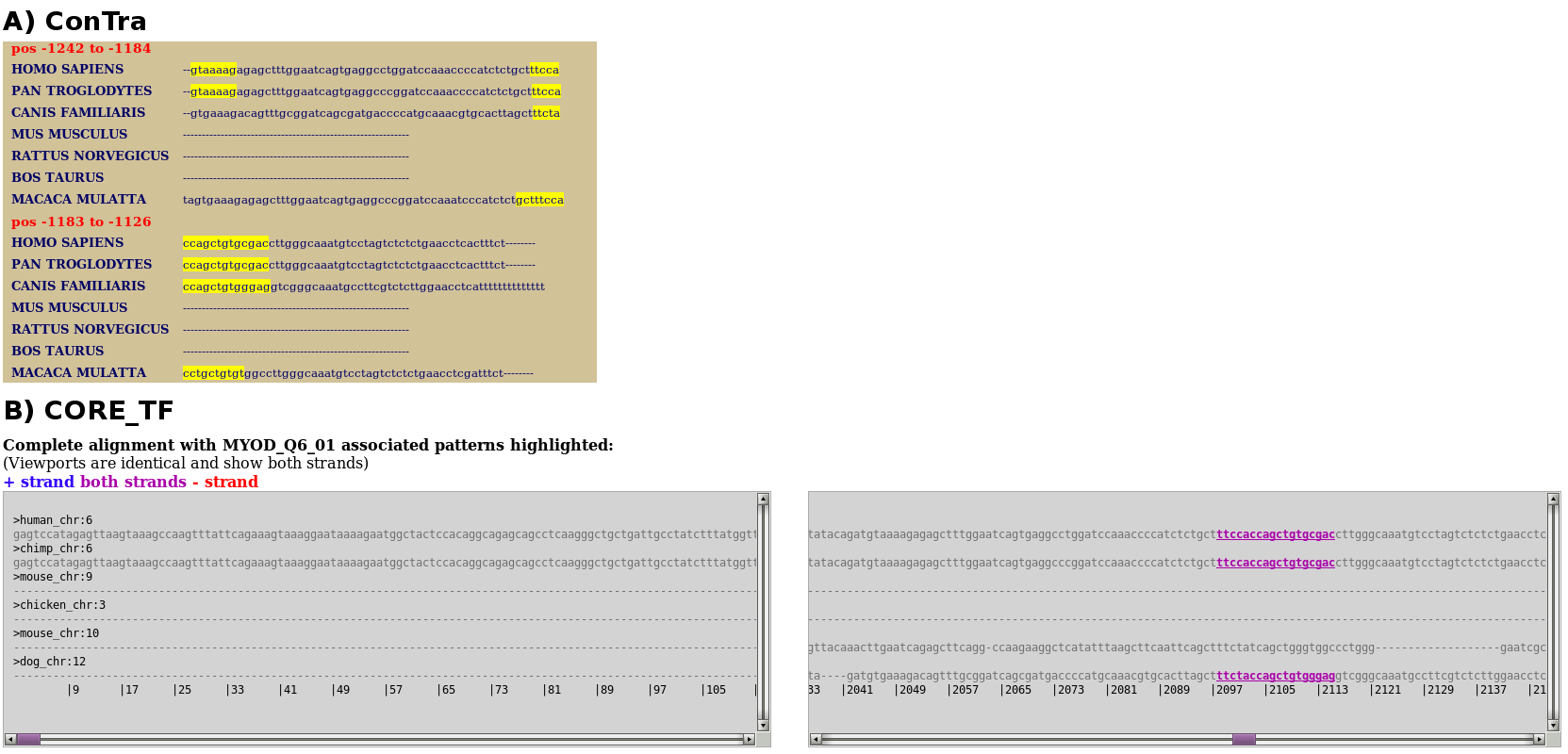

Supplement: Additional file 10 — Identifying MyoD TFBS conserved in the LAMA4 promoter with ConTra and CORE_TF. Many conserved sites were found identically between the two programs. Shown here is the most conserved TFBS found, a MyoD TFBS conserved between human, chimp, and dog in (B) CORE_TF and also macaque in (A) ConTra. Though found by both programs, CORE_TF also identifies the site is on both strands of the DNA. [file 1471-2105-9-495-S10.png]
