# Supplementary material for: CORE_TF: a user-friendly interface to identify evolutionary conserved transcription factor binding sites in sets of co-regulated genes
Source: BMC Bioinformatics. 2008 Nov 26;9:495. doi: 10.1186/1471-2105-9-495 (PMC2613159; doi:10.1186/1471-2105-9-495)
Supplement: Additional file 4 — TransFactor_LAMA4_MyoD. Set-up and data analysis of MyoD binding a LAMA4 promoter derived sequence with the TransFactor kit. [file 1471-2105-9-495-S4.pdf]

TransFactor confirmation MyoD binds the LAMA4 (ENSG00000112769:ENST00000230538) promoter

Author: Matthew Hestand
Date: September 2008

Materials:

-TransFactor Kit (Clontech product 631956)

-Oligos: (ordered from Operon, bring up in TE to 100µm)

LAMA4_MyoD_F	biotin-	tgctttcCACCAGCTGTGCgaccttg
LAMA4_MyoD_R		caaggtcgcacagctggtggaaacga
Neg_MyoD_F	biotin-	tgctttcCTCGAGGAGTGCgaccttg
Neg_MyoD_R		caaggtcgcactcctcgaggaaagca

* red highlight is the mutated nucleotides from the original target sequence

-Antibodies:

Primary: Santa Cruz MyoD (M-318): sc-760

Secondary: goat anti rabbit IgG-HRP from TransFactor Kit

-Protein:

Recombinant MyoD protein

-Plate Reader:

BIO-TEK Synergy HT

Methods:

Oligo preparation is done as:

-mix 10ul forward + 10ul reverse oligo

-place 95°C heat black 10 minutes

-cool on desktop 30 minutes

-mix 2ul with 198ul Mg to make 1 µM concentration, vortex briefly

The TransFactor Kit User Manual: V. Colorimetric TransFacor ELISA Procedure is followed with the following additions/changes:

-dilute MyoD antibody 1:100

-dilute goat anti rabbit antibody 1:1000

-step F1: after adding the TMB substrate place directly into the reader

-plate reader protocol:

1. Kinetic- 13x5minute intervals
2. Absorbance
3. Wavelength: 655nm

4. Shake 30s/read

Results:

Measurements over 5 time points:

slope: Tn-T(n-1)						
T2-T1 sample	9-26-06		9-29-06		10/06/06	
Neg_MyoD	0.01	0.008	0.003	0.005	0.027	0.028
LAMA4_MyoD	0.021	0.034	0.026	0.03	0.612	0.455
T3-T2 sample						
Neg_MyoD	0.008	0.007	0.001	0.003	0.023	0.022
LAMA4_MyoD	0.019	0.029	0.024	0.024	0.48	0.355
T4-T3 sample						
Neg_MyoD	0.007	0.006	0.001	0.001	0.017	0.02
LAMA4_MyoD	0.017	0.024	0.019	0.02	0.387	0.292
T5-T4 sample						
Neg_MyoD	0.007	0.006	0	0.003	0.017	0.017
LAMA4_MyoD	0.015	0.02	0.019	0.019	0.322	0.246

Gnumeric spreadsheet Anova single factor results:

Groups	Count	Sum	Average	Variance
measurements	48	3.756	0.07825	0.02247
sample	48	72	1.5	0.25532
day	48	96	2	0.68085

ANOVA

Source of Variation	SS	df	MS	F	P-value	F critical
Between Groups	95.4319	2	47.7160	149.324	1.5E−35	3.06029
Within Groups	45.0561	141	0.31955			
Total	140.488	143				

Conclusion:

With a p-value of 1.5E-35 there is a very significant difference in MyoD binging between the negative and target oligos. It is therefore highly likely that the target sequence is a binding site for MyoD.
